# Differences between oscillometry measurements obtained by MostGraph-01 and MasterScreen-IOS in patients with asthma

**DOI:** 10.1371/journal.pone.0309981

**Published:** 2024-09-06

**Authors:** Sonoko Harada, Norihiro Harada, Hitoshi Sasano, Yuki Tanabe, Masaki Kotajima, Yoshihiko Sato, Tomohito Takeshige, Yoko Katsura, Jun Ito, Ryo Atsuta, Hajime Kurosawa, Kazuhisa Takahashi

**Affiliations:** 1 Juntendo University Faculty of Medicine and Graduate School of Medicine, Department of Respiratory Medicine, Tokyo, Japan; 2 Juntendo University Faculty of Medicine and Graduate School of Medicine, Atopy (Allergy) Research Center, Tokyo, Japan; 3 Juntendo University Faculty of Medicine and Graduate School of Medicine, Research Institute for Diseases of Old Ages, Tokyo, Japan; 4 Clinical Engineering Unit, Juntendo University Hospital, Tokyo, Japan; 5 Department of Occupation Health, Center for Environmental Conservation and Research Safety, Tohoku University, Miyagi, Japan; Bindura University of Science Education, SOUTH AFRICA

## Abstract

**Background:**

Oscillometry devices (also termed forced oscillation technique) devices such as MasterScreen-IOS^®^ (Jaeger, Hochberg, Germany) and MostGraph-01^®^ (Chest, Tokyo, Japan) are useful for obtaining physiological assessments in patients with obstructive lung diseases, including asthma. However, as oscillometry measurements have not been fully compared between MasterScreen-IOS^®^ and MostGraph-01^®^ in patients with asthma, it is unknown whether there are differences in the measurements between the devices. This study aimed to determine whether there is any difference in oscillometry measurements obtained using the two devices in patients with asthma.

**Methods:**

Oscillometry measurements obtained using MasterScreen-IOS^®^ and MostGraph-01^®^ were retrospectively evaluated in 95 patients with asthma at Juntendo University Hospital between October 2009 and November 2009.

**Results:**

There was a strong positive correlation in the measurements between the two devices. However, the values of R5, R20, ALX and Fres were lower when measured with MostGraph-01^®^ than with MasterScreen-IOS^®^, and vice versa for the values of X5. The results were used in correction equations to convert oscillometry parameters measured using MasterScreen-IOS^®^ to those measured using MostGraph-01^®^.

**Conclusions:**

To our knowledge, this is the first report to compare MostGraph-01^®^ and MasterScreen-IOS^®^ devices using practical clinical data obtained in patients with asthma. The values obtained by both devices can be interpreted in a similar way, although there is slight variation. The conversion equations produced in this study may assist to compare the oscillometry measurements obtained by each of the two devices.

## Introduction

Measurement of respiratory resistance using oscillometry (also known as forced oscillation technique) is useful for the physiological evaluation of obstructive ventilatory disorders such as bronchial asthma [1–6]. Asthma is characterized by narrowing of the airways and hypersensitivity to airways, and spirometry is used for these objective assessments. As spirometry testing procedures are complex and depend on the respiratory effort of the subject, the facilities in which accurate spirometry measurements can be performed are limited. On the contrary, in the original method of oscillometry reported in 1956, pressure waves are generated mechanically and applied from the oral cavity to the lower respiratory tract, and airflow rate and pressure are measured over time [1]. Using this method, respiratory impedance and its components (respiratory system resistance [Rrs] and respiratory system reactance [Xrs]) can be measured while the subject breathes at rest, thus requiring no respiratory effort [1–4].

Impulse oscillometry (IOS) was developed by Michaelson in 1976 as a modified form of oscillometry and was commercialized as MasterScreen-IOS^®^ by Jaeger (Hochberg, Germany) in the 1990s [1,7]. MasterScreen-IOS^®^ is still in use worldwide. In Japan, MostGraph-01^®^ (Chest, Tokyo, Japan) was developed and commercialized as a domestic device, and is widely used in clinical settings. Both models can simultaneously obtain Rrs and Xrs in the range of 5 to 35 Hz. They are classified as auxiliary tests for spirometry and are covered by insurance for respiratory resistance measurement using the wide-range frequency oscillation method. Respiratory impedance measurements can differ between the two devices due to differences in frequency waveforms and data processing [8], and it is desirable to estimate these differences in the clinical setting. Although a previous report has measured respiratory impedance between the two devices using phantom models that imitate the airway resistance of the human body [8], abstracts from the 2012 American Academy of Allergy, Asthma and Immunology (AAAAI) annual meeting were the only sources that compared the values measured in actual adults patients with asthma. We conducted a two-month period of simultaneous use of MasterScreen-IOS^®^ and MostGraph-01^®^ devices to assess any clinical issues during the transition from MasterScreen-IOS^®^ when we introduced MostGraph-01^®^ in our hospital. Despite their continued global use, there is a scarcity of publications that compare the data obtained from these two devices, impeding effective comparisons. Therefore, we performed a retrospective analysis of historical data to compare respiratory impedance values between the two devices in a clinical setting. This study aimed to elucidate the accurate formula for converting the values of the respiratory impedance measurement between the two devices in patients with asthma.

## Methods

### Study population

Patients who visited the outpatient clinic of Juntendo University Hospital (Tokyo, Japan) between 19 October and 27 November 2009 to follow-up diagnosed asthma were enrolled in the study. Asthma was diagnosed based on a clinical history of episodic symptoms with airflow limitation and on variations in lung function, either by forced expiratory volume in one second or by peak expiratory flow (FEV_1_), in accordance with the Global Initiative for Asthma guidelines [9]. The present study was reviewed and approved by the Juntendo University Research Ethics Committee (E21-0131). Due to the retrospective nature of this study, the ethics committee waived the requirement for written informed consent. Instead, an opt-out document was made available on the hospital’s website, allowing patients to decline participation. Data access occurred between September and October 2022. Throughout and after the data collection process, the authors did not have access to any information that could identify individual participants.

### Measurement of oscillometry, respiratory function, and exhaled nitric oxide

In this study, MasterScreen-IOS® and MostGraph-01^®^ oscillometry devices were used. Each device was calibrated according to the manufacturer’s instructions before starting the measurement. Oscillometry measurements were obtained in each patient in the order of MasterScreen-IOS^®^, MostGraph-01^®^, and spirometry, and were all obtained on the same day. In both oscillometry devices, impulse signals were applied for 30 seconds and each measurement was made during tidal breathing in the sitting position. Subjects wore nose clips to prevent air leaks and supported their cheeks with both hands to reduce upper airway shunts. Airway resistance values at 5 Hz and 20 Hz (R5 and R20, respectively), the difference between R5 and R20 (R5−R20), reactance value at 5 Hz (X5), resonant frequency (Fres), and low-frequency reactance area (ALX) were measured in the inspiratory and expiratory phases, and the average of both phases was evaluated [10]. Because MasterScreen-IOS^®^ and MostGraph-01^®^ express airway resistance in different units, the values obtained by MasterScreen-IOS^®^ were converted from kPa/L/s to cmH_2_O/L/s (using 1 kPa = 10.1972 cmH_2_O) for all comparisons except Fres.

Spirometry was performed using computed spirometry (Fukuda Denshi, Tokyo, Japan). The predicted values for FEV_1_ for a Japanese population were calculated using the formula provided by the Japanese Respiratory Society [11]. FEV_1_% was calculated as FEV_1_/forced vital capacity (FVC).

The fractional exhaled levels of nitric oxide (FeNO) were measured according to the recommendations of the American Thoracic Society at a constant flow of 50 ml/s against an expiratory resistance of 10–20 cm of water with stationary NOA280i^®^ NO chemiluminescence analyzers (GE Analytical Instruments, Boulder, CO, USA) [12]. Subjects were instructed to inhale NO-free air and exhale for 10 seconds at a flow rate of 50 ml/s. The mean of 3 sufficient measurements that sustained a plateau of 10 seconds was deemed acceptable.

### Statistical analysis

Spearman’s test was used to calculate the correlation coefficient for each measurement obtained with MasterScreen-IOS^®^ and MostGraph-01^®^. The Bland–Altman method was used to evaluate the agreement between the two devices. The comparison of the Rrs and Xrs values between the two devices was performed using a Wilcoxon signed rank test. Differences were deemed statistically significant for *p* values of 0.05 or less. Statistical analyzes were performed with GraphPad Prism version 6 (GraphPad Software, San Diego, CA, USA) and JMP Pro 17 (JMP Statistical Discovery, Cary, NC, USA).

## Results

In the study, 101 patients were enrolled. However, three patients were excluded due to incomplete data, while another three patients were excluded due to the presence of COPD as a complication. Table 1 lists the demographic and clinical characteristics of the 95 patients with asthma who were evaluated. The median age (interquartile range) age was 49.0 (36.0―63.0) years and there were 61 (64.2%) women. The median duration of asthma, the median FEV_1_% and the median FeNO were 11.0 (2.0―22.0) years, 75.9 (67.3―82.2), and 43.7 (28.2―64.5) parts per billion (ppb), respectively (Table 1).

**Table 1:** Baseline characteristics of the study population.

	n = 95
Sex (M/F)	34 (35.8%)/61 (64.2%)
Age (y)	49.0 (36.0–63.0)
Age at asthma onset (y), n = 91	35.0 (22.0–50.0)
Duration of asthma (y), n = 91	11.0 (2.0−22.0)
BMI (kg/m^2^)	22.8 (20.4−25.2)
Smoking history (never/ex/current), n = 93	58 (62.3%)/27 (29.0%)/8 (8.6%)
Pack years, n = 93	0.0 (0.0−110.0)
NERD	8 (8.4%)
Atopic dermatitis	12 (12.6%)
Allergic rhinitis	46 (48.4%)
Chronic sinusitis	8 (8.4%)
ABPA	1 (1.1%)
Daily dose of ICS (FP equivalent dose, μg)	500.0 (200.0−800.0)
Oral corticosteroid treatment	14 (14.7%)
FeNO (ppb)	43.7 (28.2−64.5)
FEV_1_ (L), n = 94	2.3 ± 0.8
%FEV_1_ (predicted, %), n = 94	88.4 ± 19.9
FEV_1_% (%), n = 94	75.9 (67.3−82.2)
PEF (L/s), n = 94	7.0 ± 2.2
PEF (predicted, %), n = 94	95.6 ± 21.7
MostGraph	
R5 (cmH_2_O/L/s)	3.08 (2.05−4.84)
R20 (cmH_2_O/L/s)	2.77 ± 1.30
R5−R20 (cmH_2_O/L/s)	0.69 (0.23−1.22)
X5 (cmH_2_O/L/s)	–0.56 (–1.21 to –0.21)
ALX (cmH_2_O/L/s * Hz)	1.99 (0.68−6.14)
Fres (Hz)	8.22 (6.30−12.28)
MasterScreen	
R5 (kPa/L/s)	0.34 (0.25−0.45)
R20 (kPa/L/s)	0.29 (0.23−0.38)
R5−R20 (kPa/L/s)	0.04 (0.02−0.10)
X5 (kPa/L/s)	–0.13 (–0.19 to –0.10)
ALX (kPa/L)	0.43 (0.24−0.93)
Fres (1/s)	14.54 (11.42−18.36)
MasterScreen (Unit conversion)	
R5 (cmH_2_O/L/s)	3.47 (2.55−4.59)
R20 (cmH_2_O/L/s)	2.96 (2.35−3.87)
R5−R20 (cmH_2_O/L/s)	0.41 (0.20−1.02)
X5 (cmH_2_O/L/s)	–1.33 (–1.94 to –1.02)
ALX (cmH_2_O/L)	4.38 (2.45−9.48)

Data are presented as the number (%), mean (± SD), or median (interquartile range).

Abbreviations for all tables: ABPA, allergic bronchopulmonary aspergillosis; FEV_1_%, forced expiratory volume in 1 second/forced vital capacity; FP, fluticasone propionate; Fres, resonant frequency; FVC, forced vital capacity; NERD, nonsteroidal antiinflammatory drug-exacerbated respiratory disease.

**Fig 1:**
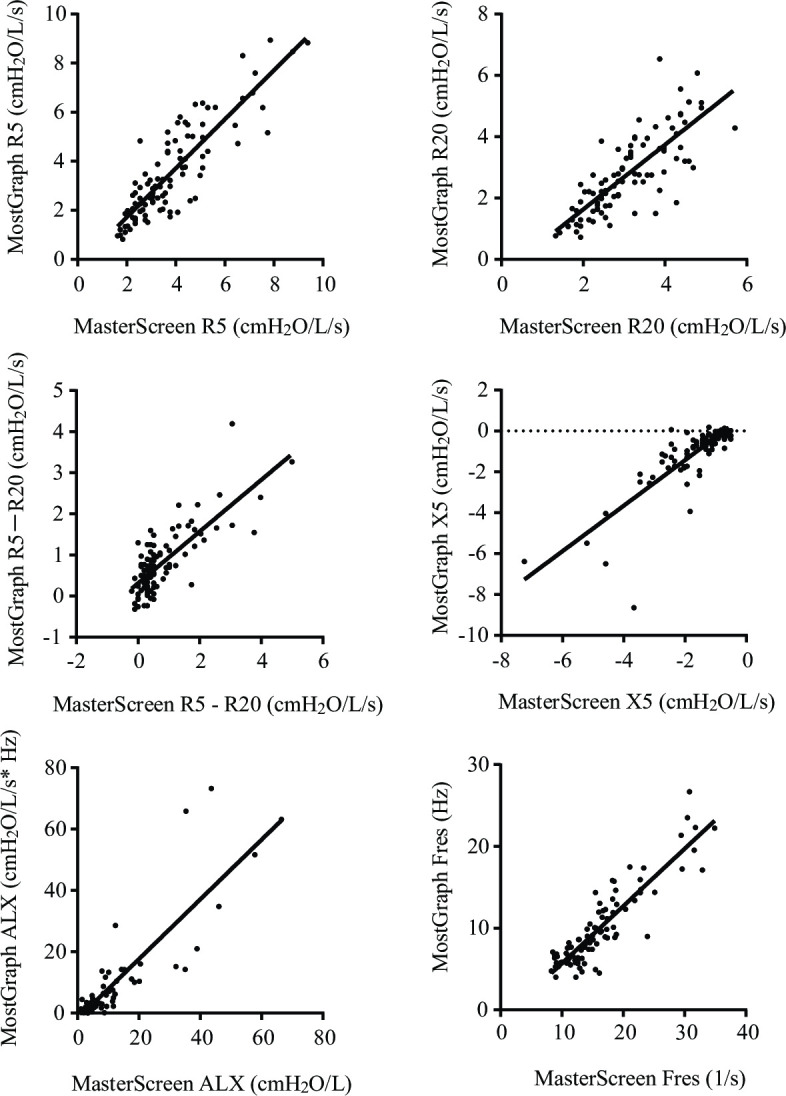
Linear regression analysis between measurements obtained with MasterScreen-IOS^®^ and MostGraph-01^®^. The coefficients of determination for the linear regression of R5, R20, R5−R20, X5, ALX, and Fres were 0.75, 0.63, 0.60, 0.68, 0.77, and 0.81, respectively. There was a strong positive correlation in the measurements of R5 (r = 0.83, p < 0.001), R20 (r = 0.82, p < 0.001), R5−R20 (r = 0.68, p <0.001), X5 (r = 0.77, p < 0.001), Fres (r = 0.84, p < 0.001), and ALX (r = 0.84, p < 0.001) between the two devices.

**Fig 2:**
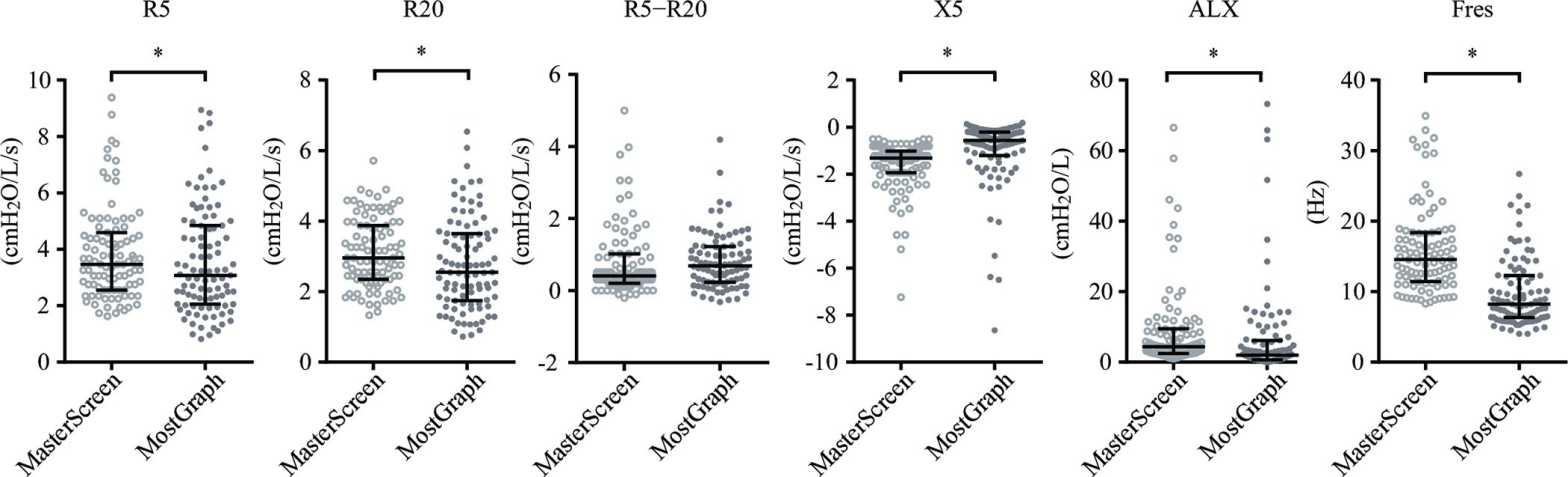
Comparison of two devices: MasterScreen-IOS^®^ and MostGraph-01^®^. The levels of R5, R20, R5−R20, Fres, and ALX measured with MostGraph-01^®^ were significantly lower than those measured with MasterScreen-IOS^®^. The bars indicate median (interquartile range). *p < 0.05.

There was a strong positive correlation in the values of R5 (r = 0.83, *p* < 0.001), R20 (r = 0.82, *p* < 0.001), R5−R20 (r = 0.68, *p* < 0.001), X5 (r = 0.77, *p* < 0.001), Fres (r = 0.84, *p* < 0.001), and ALX (r = 0.84, *p* < 0.001) values between the two devices (Fig 1). These results were similar for the inspiratory and expiratory components (S1 Fig). However, in the Rrs and Xrs measurements, the values for R5, R20, Fres, and ALX were lower with MostGraph-01^®^ than with MasterScreen-IOS^®^, and vice versa for X5 (Fig 2 and Table 2).

**Table 2:** Comparison of MostGraph^®^ and MasterScreen^®^.

n = 95	MostGraph^®^	MasterScreen^®^	*p* value	Difference
R5 (cmH_2_O/L/s)	3.08 (2.05 to 4.84)	3.47 (2.55 to 4.59)	0.004	–0.41 (–0.87 to 0.36)
R20 (cmH_2_O/L/s)	2.55 (1.74 to 3.65)	2.96 (2.35 to 3.87)	<0.001	–0.37 (–0.78 to 0.17)
R5−R20 (cmH_2_O/L/s)	0.69 (0.23 to 1.22)	0.41 (0.20 to 1.02)	0.236	0.08 (–0.29 to 0.37)
X5 (cmH_2_O/L/s)	–0.56 (–1.21 to –0.21)	–1.33 (–1.94 to –1.02)	<0.001	0.75 (0.44 to 0.97)
ALX (cmH_2_O/L)	1.99 (0.68 to 6.14)	4.38 (2.45 to 9.48)	<0.001	–2.01 (–3.48 to –1.04)
Fres (Hz)	8.22 (6.30 to 12.28)	14.54 (11.42 to 18.36)	<0.001	–5.85 (–7.59 to –4.09)

Data are presented as the median (interquartile range).

The following equations were used to convert the results obtained for each oscillometry measurement with MasterScreen-IOS^®^ to the same units as measured with MostGraph-01^®^. After conversion, the aligned units were applied to the measurements obtained with MasterScreen-IOS^®^.

R5 (MostGraph-01^®^) = 0.9986 (SE, 0.0601) × R5 (MasterScreen-IOS^®^ [kPa/L/s]) × 10.1972–0.2811 (SE, 0.2520)

R20 (MostGraph-01^®^) = 1.0541 (SE, 0.0839) × R20 (MasterScreen-IOS^®^ [kPa/L/s]) × 10.1972–0.4794 (SE, 0.2715)

R5−R20 (MostGraph-01^®^) = 0.6291 (SE, 0.0536) × R5−R20 (MasterScreen-IOS^®^ [kPa/L/s]) × 10.1972 + 0.3117 (SE, 0.0652)

X5 (MostGraph-01^®^) = 1.1148 (SE, 0.0800) × X5 (MasterScreen-IOS^®^ [kPa/L/s]) × 10.1972 + 0.8087 (SE, 0.1580)

ALX (MostGraph-01^®^) = 0.9735 (SE, 0.0549) × ALX (MasterScreen-IOS^®^ [kPa/s]) × 10.1972–1.8862 (SE, 0.8249)

Fres (MostGraph-01^®^) = 0.7001 (SE, 0.0357) × Fres (MasterScreen-IOS^®^ [1/s])– 1.3083 (SE, 0.6076)

The coefficients of determination for the linear regression of R5, R20, R5−R20, X5, ALX, and Fres were 0.75, 0.63, 0.60, 0.68, 0.77, and 0.81, respectively.

**Fig 3:**
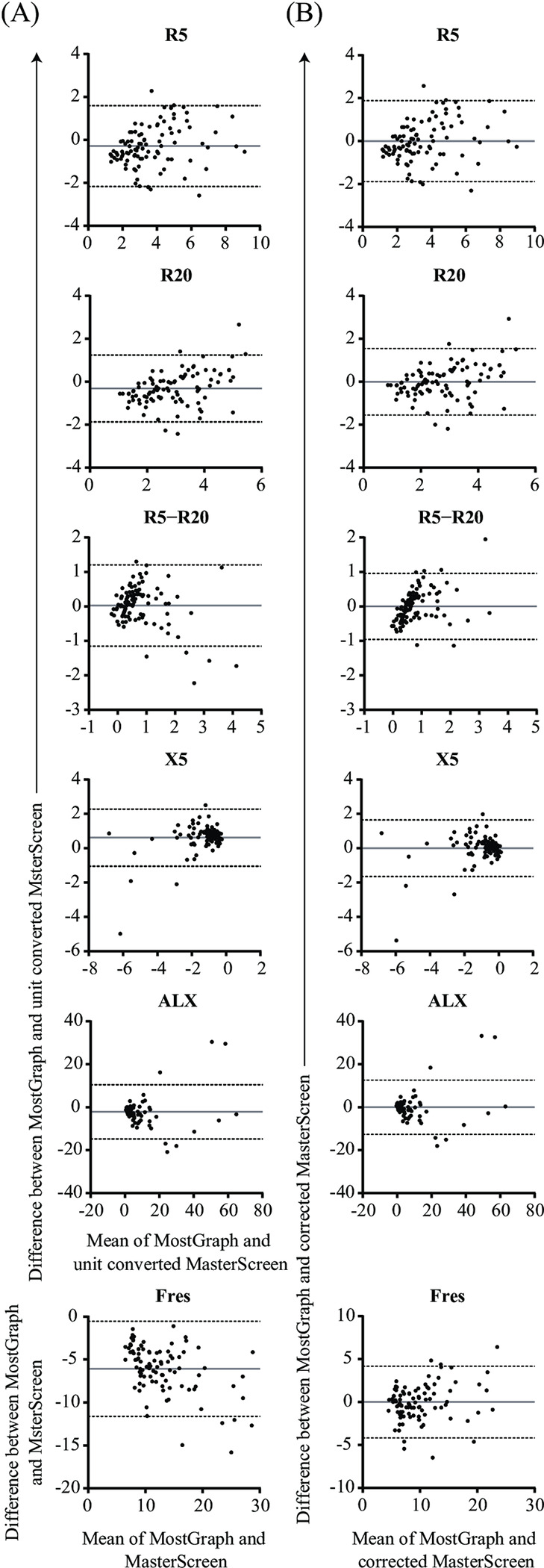
Bland–Altman plots for the evaluation of agreement between the two analyzers. The mean values obtained with the two devices were plotted on the x-axis and the difference in each parameter level (MostGraph-01^®^ value–the value with converted unit of MasterScreen-IOS^®^) was plotted on the y-axis, except Fres (A). Because Fres has the same unit for both devices, the difference between the measured values was plotted. After applying the conversion equation to correct the levels of each value measured with MasterScreen-IOS^®^, the mean of each value obtained with MostGraph-01^®^ and the corrected value measured with MasterScreen-IOS^®^ were plotted on the x-axis, and the difference in levels (MostGraph-01^®^ –corrected MasterScreen-IOS^®^) was plotted on the y-axis (B). The solid gray line indicates the mean of difference, and the dotted lines are the 95% limits of agreement.

In the consistency evaluation between the two devices, the Bland–Altman plot demonstrated a proportional error between MostGraph-01^®^ and units converted to MasterScreen-IOS^®^ (Fig 3A). Even after conversion to align the units with those of MasterScreen-IOS^®^, there was a proportional error, but the mean difference between the two measurements was zero and the bias was corrected (Fig 3B and Table 3). These findings suggest that although most of the technical parameters were similar between the two devices, the correction equation calculated in this study assisted in the interpretation of oscillometry measurements obtained using the devices.

**Table 3:** The limits of agreement of the difference between Masterscreen^®^ and Mostgraph^®^.

	Before applying the conversion equation			
	Mean of difference (95%CI)	SD of difference	Lower LOA (95%CI)	Upper LOA (95%CI)
R5	-0.29 (-0.48 to -0.09)	0.96	-2.17 (-2.51 to -1.83)	1.60 (1.26 to 1.93)
R20	-0.31 (-0.47 to -0.15)	0.79	-1.87 (-2.14 to -1.59)	1.24 (0.97 to 1.52)
R5−R20	0.026 (-0.097 to 0.15)	0.60	-1.15 (-1.36 to -0.94)	1.21 (0.99 to 1.41)
X5	0.62 (0.45 to 0.79)	0.85	-1.04 (-1.33 to -0.75)	2.28 (1.98 to 2.58)
ALX	-2.12 (-3.44 to -0.81)	6.45	-14.76 (-17.01 to -12.50)	10.51 (8.26 to 12.76)
Fres	-6.07 (-6.65 to -5.50)	2.82	-11.60 (-12.59 to -10.62)	-0.54 (-1.52 to 0.45)
	After applying the conversion equation			
	Mean of difference (95%CI)	SD of difference	Lower LOA (95%CI)	Upper LOA (95%CI)
R5	0.00 (-0.20 to 0.20)	0.96	-1.88 (-2.22 to -1.55)	1.88 (1.55 to 2.22)
R20	0.00 (-0.16 to 0.16)	0.79	-1.55 (-1.83 to -1.27)	1.55 (1.27 to 1.83)
R5−R20	-0.00 (-1.00 to 1.00)	0.49	-0.96 (-1.13 to -0.79)	0.96 (0.79 to 1.13)
X5	-0.00 (-0.17 to 0.17)	0.84	-1.64 (-1.94 to -1.35)	1.64 (1.35 to 1.94)
ALX	0.00 (-1.31 to 1.31)	6.44	-12.62 (-14.87 to -10.37)	12.62 (10.37 to 14.87)
Fres	-0.00 (-0.43 to 0.43)	2.13	-4.17 (-4.91 to -3.43)	4.17 (3.43 to 4.91)

## Discussion

This study uses practical clinical data from asthma patients to compare the values obtained by the MasterScreen-IOS^®^ and MostGraph-01^®^ oscillometry devices. Although the results obtained by the devices can be interpreted similarly, there is a slight deviation in values, and therefore the application of the equation used in this study would allow a more accurate evaluation for patients with asthma.

MasterScreen-IOS^®^ is in widespread use worldwide, whereas MostGraph-01^®^ was released in 2009 and is popular in Japan but is in limited use worldwide. In terms of hardware and software, oscillometry devices differ in the oscillation signal waveforms used and in data processing. MasterScreen-IOS^®^ alternates between positive and negative impulse signals regardless of the direction of flow in the airway opening. On the contrary, the direction of the pulse signal in MostGraph-01^®^ is matched to flow direction; i.e., inspiration or exhalation. A previous study has reported that impulse signals used with MasterScreen-IOS^®^ produce respiratory impedance values similar (but not identical) to those provided by oscillometry using pseudorandom noise signals [2]. Furthermore, a basic study performed using phantom models that mimic the resistance of the human body airways demonstrated differences in resistance values and frequency dependence for resistance between the same oscillometry devices used in the present study [8]. In agreement with the present results, they found that the reactance values measured by MostGraph-01^®^ were higher than those measured by MasterScreen-IOS^®^ [8]. There may be some differences in the oscillometry measurements between the two devices and between the two types of signals, and the oscillometry devices did not always produce identical measurement results consistently [2,8,13]. There is limited awareness of the measurements distinctions between MasterScreen-IOS^®^ and MostGraph-01^®^ when used in actual patients. It is important to estimate the differences when comparing clinical results that have been obtained using two different devices. Until now, the investigation examining disparities in measured oscillometry during clinical practice between the two devices was only presented at the 2012 AAAAI annual meeting abstract, and therefore it is necessary to investigate practical oscillometry parameters at various frequencies in patients with various lung diseases. Although several clinical studies have used MasterScreen-IOS^®^ or MostGraph-01^®^ to investigate the actual oscillometry measurements of real patients with obstructive airway disease in clinical practice, none have compared the two devices in these studies [14–18]. Therefore, the present result of a slight difference between the two devices is valuable because it has been obtained using practical clinical data from asthma patients.

The study has some limitations. It was performed as a retrospective analysis in a single center. As the target subjects were limited to patients with asthma, further studies are needed to investigate whether similar results would be obtained in healthy subjects, and in patients with chronic obstructive pulmonary disease and other respiratory diseases. As the order of measurement was fixed as MasterScreen-IOS^®^ followed by MostGraph-01® and no crossover was performed, we cannot rule out that the order of testing could have affected the results. Despite these limitations, the present study allowed clarification of differences between the two oscillometry devices using data from asthma patients in clinical practice, which has previously been difficult due to the inaccessibility of the data. Furthermore, the present study has provided the correction equation to convert oscillometry measurements obtained with MasterScreen-IOS^®^ to those measured with MostGraph-01^®^. This equation can facilitate the comparison of measured oscillometry parameters between the two devices in clinical practice and also in epidemiological studies.

## Conclusions

This study shows a strong positive correlation in asthma patient oscillometry measurements between MasterScreen-IOS^®^ and MostGraph-01^®^, and is the first to identify slight differences in measured values between them. In addition, the correction equation used in the present study to convert the oscillometry measurements obtained with MasterScreen-IOS^®^ to those measured with MostGraph-01^®^ increases the accuracy of the comparison of oscillometry parameters measured with two different devices.

## Supporting information

S1 Fig (TIF)

## Abbreviations

ALX: low-frequency reactance area
FEV_1_%: forced expiratory volume in 1 second/forced vital capacity
Fres: resonant frequency
FVC: forced vital capacity
IOS: impulse oscillometry
R5: resistance value at 5Hz
R20: resistance value at 20Hz
Rrs: respiratory system resistance
X5: reactance at 5Hz
Xrs: respiratory system reactance
