# Supplementary figures and images for: Differences between oscillometry measurements obtained by MostGraph-01 and MasterScreen-IOS in patients with asthma
Source: PLoS One. 2024 Sep 6;19(9):e0309981. doi: 10.1371/journal.pone.0309981 (PMC11379245; doi:10.1371/journal.pone.0309981)

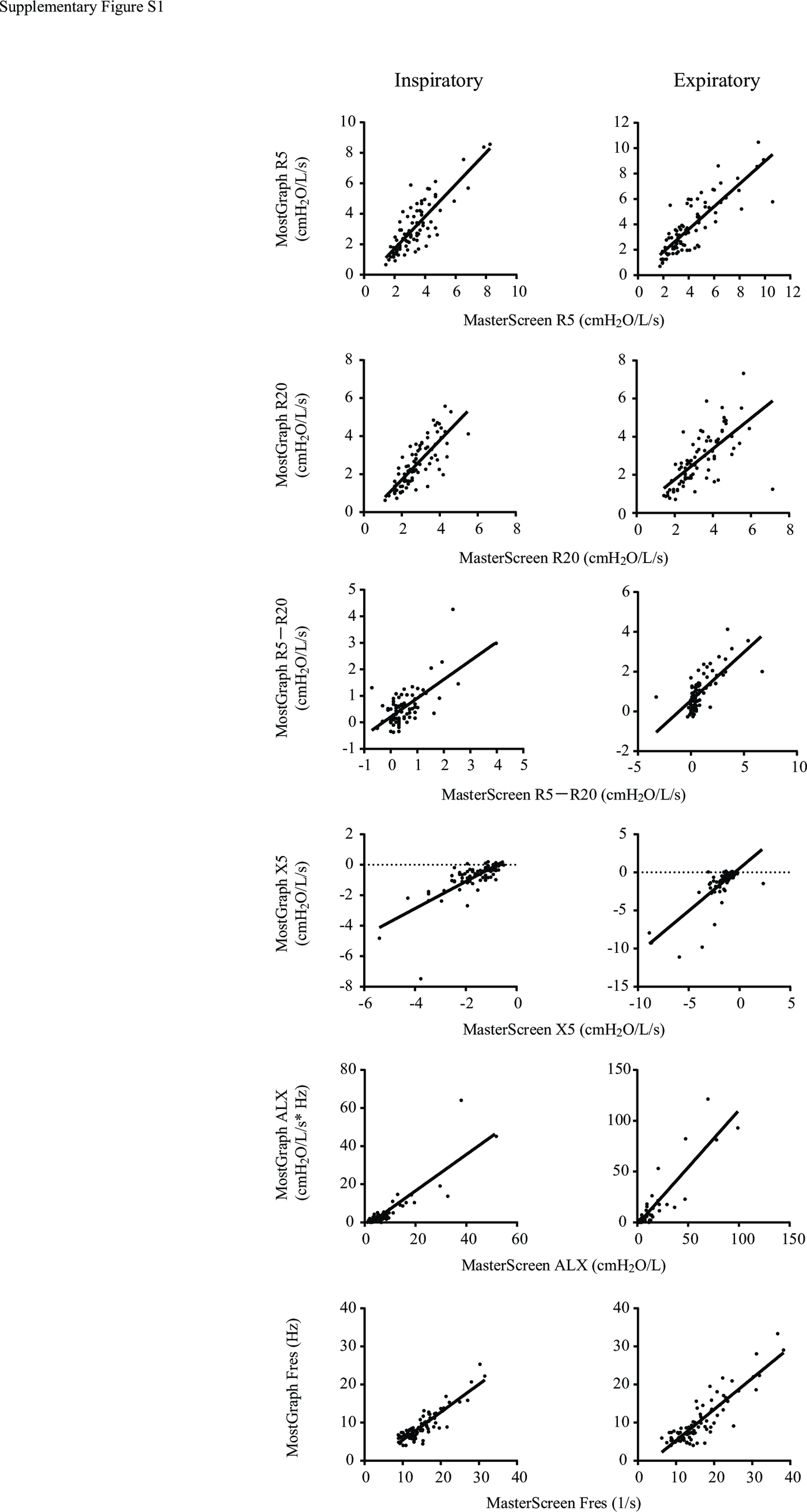

Supplement: S1 Fig — (TIF) [file pone.0309981.s001.tif]
